# 西妥昔单抗对非小细胞肺癌细胞的体外抑制作用

**DOI:** 10.3779/j.issn.1009-3419.2010.08.03

**Published:** 2010-08-20

**Authors:** 真 陈, 智伟 陈

**Affiliations:** 1 200438 上海，上海市杨浦区市东医院病理科 Department of Pathology, Shidong Hospital, Shanghai 200438, China; 2 200030 上海，上海交通大学附属胸科医院，上海市肺部肿瘤临床医学中心 Shanghai Lung Tumor Clinical Medical Center, Shanghai Chest Hospital Affiliated to Shanghai Jiaotong University, Shanghai 200030, China

**Keywords:** 西妥昔单抗, 肺肿瘤, 细胞增殖, 凋亡, 表皮生长因子受体, Cetuximab, Lung neoplasms, Cell proliferation, Apoptosis, Epidermal growth factor receptor

## Abstract

**背景与目的:**

目前抗EGFR单抗——西妥昔单抗在肺癌的临床运用越来越广泛。本研究旨在探讨西妥昔单抗对人肺癌细胞株（A549、H460、H1299、SPC-A-1）的体外作用。

**方法:**

我们选用浓度递增的西妥昔单抗（1 nmol/mL-625 nmol/mL）作用于A549、SPC-A-1、H460、H1229四株细胞株，采用CCK8测定各组细胞增殖抑制情况，选择A549、SPC-A-1细胞株用PI标记应后用流式细胞技术观察细胞凋亡情况，并采用免疫蛋白印迹法检测药物处理后A549细胞EGFR信号转导通路关键酶的表达。

**结果:**

西妥昔单抗对A549、H460、H1299、SPC-A-1细胞的作用均呈时间和浓度依赖性，作用后细胞的凋亡现象明显，同时细胞增殖均不同程度受到抑制；通过Western blot检测发现p-AKT、p-EGFR、p-MAPK蛋白表达量较对照组明显下降。

**结论:**

西妥昔单抗在体外对肺癌细胞的增殖具有一定的抑制作用，其作用可能与进一步下调了活化的EGFR信号转导通路关键酶的表达有关。

## Abstract (en)

**Background and objective:**

The epidermal growth factor receptor (EGFR) monoclonal antibody cetuximab has been used widely in non-small cell lung cancer patients. The aim of this study is to explore the effect of lung cancer cells (A549, H460, H1299, SPC-A-1) which were treated by cetuximab *in vitro*.

**Methods:**

We studied the effects of increasing concentrations of cetuximab (1 nmol/L-625 nmol/L) in four human lung cancer cell lines (A549, SPC-A-1, H460, H1229). CCK8 measured the inhibition of cell proliferation in each group. A549, SPC-A-1 were marked by PI and the statuses of apoptosis were observed. Western blot were used to detect the proliferation-related signaling protein and apoptosis-related protein in A549.

**Results:**

The treatment with cetuximab resulted in the effect on cell proliferation and apoptosis in a time- and dosedependent manner. The expression of activated key enzymes (p-AKT, p-EGFR, p-MAPK) in EGFR signaling transduction pathway were down-regulated more obviously.

**Conclusion:**

Cetuximab is an effective targeted drug in the treatment of lung cancer cell lines, tissues, most likely to contribute to the inhibition of key enzymes in EGFR signaling transduction pathway.

肺癌分子靶向治疗常用的治疗靶点有：细胞受体、信号传导和抗血管生成等，其中表皮生长因子受体（epidermal growth factor receptor, EGFR）是目前最为主要的靶点。目前已被美国食品药品监督管理局（Food and Drug Administration, FDA）批准用于临床治疗的EGFR家族特异性靶向药物主要分为两大类：一类是小分子EGFR酪氨酸激酶抑制剂（tyrosine kinase inhibitor, TKI），如吉非替尼（gef itinib）和厄洛替尼（erlotinib）; 另一类是人源化单克隆抗体，如EGFR特异性抗体西妥昔单抗（cetuxmab）和HER2特异性抗体赫赛汀（trastuzumab）。针对EGFR进行抗肿瘤治疗的单克隆抗体可阻断配体与EGFR的结合。这些抗体能够识别受体的胞外结构域，竞争与配体的结合位点，抑制EGFR的二聚化并下调细胞表面EGFR的数量^[1]^。有研究者^[2]^认为EGFRTKI的疗效受到肿瘤组织*EGFR*基因突变的影响，没有发生突变的患者可能表现为无效; 而*EGFR*基因突变并不影响EGFR单抗的疗效，因此受到人们的关注。

已有相关文献报道了西妥昔单抗应用于肺癌治疗的临床研究，其中不乏Ⅲ期临床研究证据。本研究的目的在于探讨运用西妥昔单抗对人肺癌细胞和增殖的体外抑制作用，对EGFR信号转导通路关键酶：磷酸化EGFR（p-EGFR）、磷酸化MAPK（p-MAPK）、磷酸化AKT（p-AKT）蛋白表达水平的影响，探讨其作用的分子机制。

## 1 材料与方法

### 1.1 材料

人肺癌细胞株A549、SPC-A-1、H460、H1229购自中国科学院上海细胞所，西妥昔单抗由德国默克公司提供。小鼠抗erbB-2、c-myc抗体、荧光标记的抗小鼠抗体购自北京中山公司。活细胞计数试剂盒-CCK-8（cell counting kit-8）购自日本株式会社同仁化学研究所。

### 1.2 细胞培养和分组

细胞于含10%小牛血清的DMEM培养液中常规培养。4株细胞铺96孔板，用西妥昔单抗分别以起始浓度1 nmol/L五倍的浓度递增，进行细胞药物干预，同时设置好非药空白对照。

### 1.3 细胞增殖检测

通过CCK8实验法分别测定4株人肺癌细胞株在不同药物浓度作用下的抑制率药物干预72 h后分别做CCK8实验，在每个孔内加入10 μL的CCK-8试剂。把培养板放在培养箱内培养1 h-4 h。在450 nm波长处测定吸光度，参比波长为600 nm。各药物浓度3个重复，分别求出对各细胞的抑制率：肿瘤细胞抑制率=（1-实验组的吸光度/对照组的吸光度）×100%，测定各组细胞72 h增殖抑制情况。

### 1.4 细胞凋亡检测

对选定的经西妥昔单抗分别作用的A549、SPC-A-1两株细胞，利用流式细胞仪方法检测经西妥昔单抗处理后细胞所处的分裂周期。干预72 h后观察并对3组细胞状态显微拍照，碘化吡啶（PI）标记细胞凋亡情况，各组各药浓度做一次PI凋亡流式细胞仪检测。

### 1.5 细胞迁移能力检测

收集细胞，根据凋亡和迁移能力程度的改变，检测相应的信号通路蛋白分子在蛋白水平的变化。Western blot检测SPC-A-1细胞信号通路蛋白相应的磷酸化蛋白p-AKT、p-EGFR、p-MAPK的表达，以β-actin的水平作为等量蛋白质上样，对照空白对照组，每个样本重复3遍。取50 μg蛋白质样品进行SDSPAGE电泳，转至硝酸纤维膜上; 室温封闭2 h后，用含0.05%Tween220的TBS缓冲液漂洗3次，每次10 min; 加入相应的单克隆抗体1:800，4 ℃孵育过夜，TBST漂洗3次后加入相应的辣根过氧化物酶标记的二抗1:5 000，37 ℃摇床温育2 h，ECL显色系统显色。最后经ECL系统曝光显影，通过凝胶分析系统分析蛋白的表达。

### 1.6 统计学分析

统计使用SPSS 13. 0统计软件，数据以Mean±SD表示，统计学分析采用*t*检验和单因素方差分析进行，以*P* < 0.05为有统计学差异。

## 2 结果

### 2.1 不同浓度西妥昔单抗对A549、SPC-A-1、H460、H1229细胞株增殖的抑制

在药物作用下，4种细胞株的增殖均有下降，受到明显的抑制，并随着药物浓度的增高抑制效果增强（图 1）。

**1:**
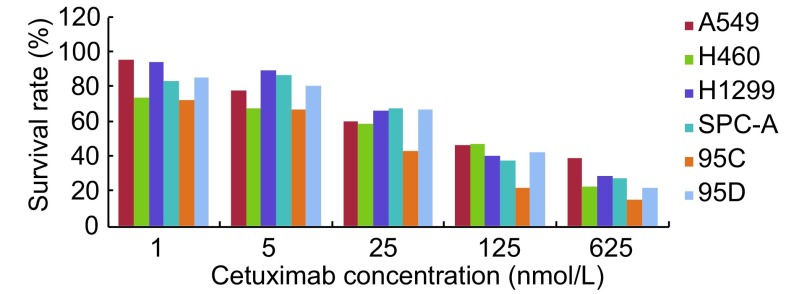
西妥昔单抗作用后对各细胞株（A549、H460、H1299、SPC-A-1、95C和95D）的增殖抑制结果 The inhibitory effects of cetuximab on proliferation of human lung cancer cells (A549, H1299, SPC-A-1, H460, 95C and 95D) treated by different concentrations of cetuximab

### 2.2 不同浓度西妥昔单抗对A549、SPC-A-1、H460、H1229细胞株凋亡的影响

药物处理细胞后，多数细胞被阻断于G_1_期，并随着药物浓度的增加被阻断于G_1_期的细胞增多。药物连续处理细胞株后，药物组细胞的凋亡率也均高于空白对照组（图 2）。

**2:**
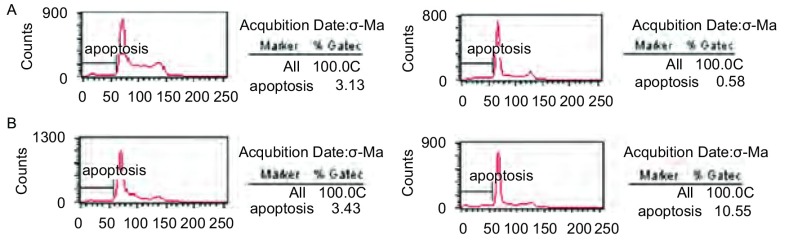
西妥昔单抗作用后A549和SPC-A-1细胞凋亡图。A：西妥昔单抗作用后A549细胞凋亡图; B：西妥昔单抗作用后SPC-A-1细胞凋亡图。 The apoptosis rates of A549 and SPC-A-1 cells treated with cetuximab. A: The apoptosis rate of A549 cells treated with cetuximab; B: The apoptosis rate of SPC-A-1 cells treated with cetuximab.

### 2.3 西妥昔单抗作用对EGFR信号通路蛋白的影响

虞永峰等^[3]^发现SPC-A-1细胞株EGFR表达情况较好。我们选择Western blot检测SPC-A-1细胞信号通路蛋白相应的磷酸化蛋白p-AKT、p-EGFR、p-MAPK的表达。结果显示：与对照组相比，西妥昔单抗作用SPC-A-1可明显降低p-AKT、p-EGFR、p-MAPK蛋白表达水平，药物作用与肺癌细胞信号通路蛋白表达抑制明显相关（*P* < 0.001）（图 3）。

**3:**
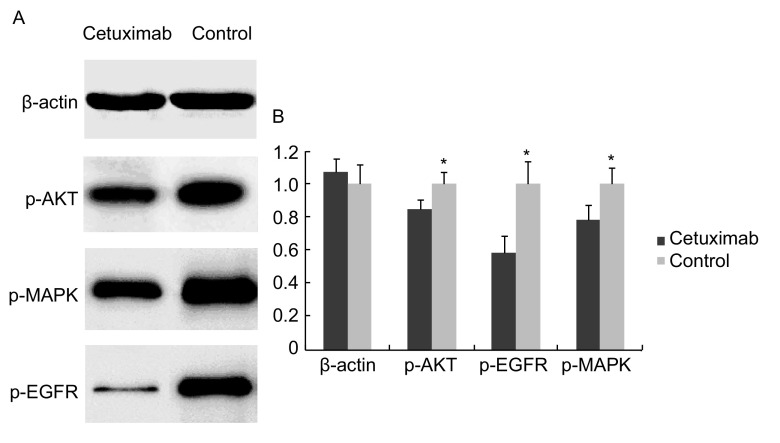
西妥昔单抗作用后SPC-A-1细胞p-AKT、p-EGFR、p-MAPK的表达下调。A：西妥昔单抗作用后SPC-A-1细胞p-AKT、p-EGFR、p-MAPK的Western blot电泳图，以*β*-actin为内对照; B：直方图显示西妥昔单抗作用SPC-A-1能明显降低p-AKT、p-EGFR、p-MAPK蛋白表达水平。^*^P < 0.05。 The expressions of p-AKT, p-EGFR, p-MAPK were downregulated in SPC-A-1 cell treated with cetuximab. A: The Western blot images of p-AKT, p-EGFR, p-MAPK in SPC-A-1 treated with cetuximab. *β*-actin served as internal control; B: The expressions of p-AKT, p-EGFR, p-MAPK were reduced significantly by cetuximab in SPC-A-1 cells than internal control. ^*^P < 0.05.

## 3 讨论

西妥昔单抗是一个特异性针对EGFR的IgG1单克隆抗体，与非活化的EGFR的胞外结构域结合，且亲和力远高于内源性配体，可以竞争性阻断配体与受体的结合，从而阻断内源性配体介导的EGFR酪氨酸激酶活化^[4]^。目前大量临床研究^[5-8]^已证实西妥昔单抗在转移性结直肠癌以及头颈部鳞状细胞癌中疗效确切。

单克隆抗体西妥昔单抗在肺癌治疗中的运用越来越广泛。2008年美国临床肿瘤年会中报道的FLEX Ⅲ期随对照研究^[9]^显示，西妥昔单抗联合化疗一线治疗晚期NSCLC可以显著延长各病理类型患者的中位生存期（11.3个月*vs* 10.1个月）与1年生存率（47% *vs* 42%）。FLEX试验是第一个证明EGFR抑制剂靶向药物与化疗联用可以延长生存的临床研究，目前被2009版NCCN指南推荐作为晚期NSCLC患者的一线治疗。

西妥昔单抗作用机制为：与EGF、TGF等配体竞争EGFR分子上的特殊结合位点，阻断配体对受体的激动作用，并通过EGFR的内吞、失活，下调其在胞膜的表达水平，还可激活抗体依赖的细胞毒作用（antibodydependent cell-mediated cytotoxicity, ADCC），产生进一步的细胞杀伤效应^[10]^。本研究结果显示西妥昔单抗对4株细胞株都表现出一定的体外增殖抑制作用，其抑制作用呈时间和浓度依赖性。随着作用时间的延长，其对细胞的增殖抑制作用逐渐增强。

我们采用Westem blot法检测经西妥昔单抗处理SPC-A-1可明显降低p-AKT、p-EGFR、p-MAPK蛋白表达水平，药物作用与肺癌细胞信号通路蛋白表达抑制明显相关进一步下调了其下游信号转导通路中活化的关键酶蛋白的表达，从而体现了明显的作用。

在人肺癌细胞系的体外作用中，西妥昔单抗具有一定的抑瘤效应，其作用的分子机制是进一步下调了活化的EGFR信号转导通路关键酶的表达，这为进一步的动物实验和临床研究、临床用药提供了一定的指导作用。EGFR单克隆抗体靶向治疗肺癌具有良好的应用前景，值得进一步的研究。
